# Pathology and Therapeutics

**Published:** 1859-11

**Authors:** Emil Fischer


### PATHOLOGY AND THERAPEUTICS.

BY EMIL FISCHER, M.D.

### I.—*On Fatal Steatosis* (*Fatty Degeneration*) *of the Liver and Kidneys.* By PROFESSOR ROKITANSKY.

Prof. Rokitansky states that several cases of death occurring after an apparently acute disease, had recently attracted his attention, first, on account of the post-mortem appearances they presented; and, secondly, from the circumstance that acute disease and its symptoms were evidently owing to uræmia which had developed itself slowly and imperceptibly, but had suddenly assumed a very violent character. This uræmia was caused by a disease of the kidneys, which undoubtedly had the same relation to a disease of the liver as the peculiar condition of the kidneys observed in acute atrophy of the liver bears to that affection. The very numerous cases which the author had examined since the year 1839 showed that in acute atrophy of the liver the kidneys always undergo a change essentially analogous to that of the liver; but that this change is not necessarily proportionate to the degree of destruction to which the substance of the liver had been subjected; it consists in a flaccid, collapsed, softened, and even diffluent state of the renal tissue, accompanied by icteric discoloration, anæmia, and ecchymoses, particularly of the cortical substance. Further examination reveals a molecular disintegration of the epithelial cells, accumulation of fat in some of them, the cells being swelled in consequence of this accumulation, and, finally, the presence of fat in large quantity. This condition, and the diminished secretion of urine attending the disease, render it highly probable that a corresponding degree of uræmia coexists in such cases. Frerichs (*Klinik der Leberkrankheiten*, 1858,) expresses himself in the following manner, referring to the observations made by Späth, (*Wiener Medizinische Wochenschrift*, 1854:) "There is not as much attention paid to the kidneys as they deserve. Besides the deposit of pigment owing to the icterus, I found that the epithelial cells of the glands were infiltrated with granular matter, and, for the most part, in a state of fatty degeneration, and that the tissue itself was flaccid and soft. These cases occurred mostly among pregnant women, in whom Späth has made the same observation. Whether this renal disease is found in the generality of cases, I leave undecided; the peculiar change of the urine, the disappearance of urea from it, and the accumulation of the latter in the blood—further, the transitory albuminuria, etc., point, at any rate, to a coexisting affection of the kidneys."

In the present state of our knowledge, the most plausible view about the relation of the disease of the liver to that of the kidneys seems to be that the latter is dependent upon, or secondary to, the hepatic affection.

The cases referred to in the beginning of this memoir consisted in *steatosis of the liver, accompanied by a high degree of steatosis of the kidneys;* their importance rests upon the possibility of proving them to be parallel to the cases of acute atrophy of the liver and the analogous renal affection which coexists with it; our task consists, therefore, in showing that the steatosis of the liver is the primary, the steatosis of the kidneys, however, the secondary disease, and that the symptoms, as well as the fatal termination, are owing to uræmia, (to the disease of the kidneys.) The material afforded by the observation of cases is unsatisfactory, owing to the circumstances under which the observations were made; and although the post-mortem examination furnishes very useful and complete data, we can presume but an attempt at the solution of the question. It will, nevertheless, hardly remain doubtful that there exists a steatosis of the liver which leads to a fatal termination by means of an analogous degeneration of the kidneys. The only statements which have reference to our subject are found in *Wunderlich's Hand-book;* in treating of fatty liver, (vol. iii. part 3, p. 327,) he says, for instance: "Only when the disease is rapidly developed, pain, fever, and even serious cerebral symptoms, with delirium, sopor, prostration, and a speedy dissolution are observed." And in speaking of fatty degeneration of the kidneys, (ibid., p. 453,) he remarks that it sometimes accompanies fatty degeneration of the liver.

After these preliminaries the author communicates three cases observed by him, all of which present one and the same post-mortem appearance; also the symptoms noticed at the sick-bed coincide with each other. After having carefully examined these cases, the author continues his attempt at the solution of the question proposed above, as follows:—

"It is evident that in our cases we have not to deal with that steatosis of the liver which occurs so commonly in the course of consuming suppurative processes, but with fatty livers, as they not rarely develop themselves to a high degree at the side of an abundant formation of fat in the areolar tissue, without the disease being always attributable to gross feeding. Further, it must be understood that the steatosis of the kidneys does not present that form which results from Bright's disease. Neither do the post-mortem appearances of the kidneys exhibit all those characteristics which mark this consecutive degeneration of the renal epithelium, nor do the symptoms during life indicate that Bright's disease and albuminuria existed and had passed, with the steatosis of the kidneys, into a second degenerative stage. Under these circumstances it can hardly admit of doubt, that the steatosis of the kidneys supervened secondarily upon the primary steatosis of the liver; and if we consider the high degree of steatosis of the kidneys, and the deficiency of urine in the urinary passages on one side, and the very slight manifestation of the signs of cholæmia on the other side, it is just as little to be doubted that the symptoms of the disease, appearing suddenly and increasing rapidly until death, are owing to the suppression of the urinary secretion—that is, to uræmia.

There exists thus a steatosis of the liver, occurring in individuals inclined to the formation of fat, to which sooner or later a steatosis of the kidneys is added, both which diseases attain slowly and imperceptibly so high a degree that, finally, a cessation of the biliary and urinary secretion supervenes, and, after a slight degree of icterus, death rapidly sets in from uræmia and a hemorrhagic decomposition of the blood. The similarity of this combined disease of the liver and kidneys with acute atrophy of the liver and the degeneration of the kidneys supervening upon it, is unmistakable; it will become still more evident, if it can be demonstrated that in the latter disease the affection of the kidneys never fails to make its appearance in a corresponding degree, and that the symptoms attending it are, for the most part, of a uræmic character.

The cases reported by the author have arrested his attention, not only on account of the marked pathological changes of the liver and kidneys, but also on account of the individuality of the patients concerned. He does not doubt that cases of the same kind have occurred before, but have escaped notice and recognition, and that especially some of those not rare cases, where neglected drunkards perished unnoticed and without medical care, or died after a short sojourn in the hospital, under sopor and delirium, belonged to this class.—(*Zeitschrift der K. K. Gesellschaft der Aerzte zu Wien*, 8 August, 1859.)

II.—*On Abscess in the Anterior Mediastinum.* By Prof. Guntner.

Prof. Güntner, having observed four cases of abscess in the anterior mediastinum, gives the following description of this disease:—

Before the swelling appears, there exist, for a longer or shorter period of time, symptoms which are referable to compression of the parts forming the posterior boundary of the anterior mediastinum; they consist of oppression of the chest, difficulty of breathing, dull and oppressive pain behind the sternum, cough, sometimes a tickling sensation in the larynx with inclination to vomit, palpitation of the heart, cyanosis of the face, and fever of various intensity. The diagnosis becomes certain only with the appearance of the swelling; in the majority of cases the swelling comes on quickly, extends over a considerable space, is in the beginning indifferent, but little painful, and exhibits no inflammatory character, or inclination to open; at the same time the subjective symptoms decrease in intensity. But as the exudation generally proceeds to softening, the swelling increases in height, becomes more concentrated, and, under inflammatory symptoms, prepares itself to burst; the fever remains a constant companion, and exhibits either a remittent or intermittent character. Perforation takes place most commonly on the left side, at the junction of the second rib with the sternum. Besides the influence of cold, no important cause could be ascertained to give rise to the disease, and it must, therefore, be regarded as an idiopathic inflammation of the cellular tissue in the anterior mediastinum.

The prognosis of the disease depends upon the rapidity of its course, the amount of exudation, and the quickness with which the latter undergoes softening. The dangers to be feared are perforation into the cavity of the pleura, then secondary pleurisy, pericarditis, pyæmia, and sinking of the system from long-continued suppuration.

The treatment is partly general, partly local. The general treatment can be but symptomatic, slightly antiphlogistic in the beginning, and afterwards tonic. The local treatment consists at first in the application of dry heat; later, when the formation of an abscess becomes distinct, an opening should be made as early as possible in the spot where the tension is greatest. The incision should be small, as it enlarges itself by subsequent ulceration. Free exit should be given to the pus; the dressing, consisting of fenestrated lint and cotton with cerate, should be often changed, and absolute rest should be enjoined. When the swelling and suppuration have decreased, the œdema and fever have ceased, and the patient can move without pain, he may be permitted to enjoy the fresh air. Only in cases where a dangerous pressure is exerted in consequence of the retention of pus, it may become necessary to increase the depth of the incision, trephine the sternum, resect the ribs, etc.; but patients are rarely inclined to subject themselves to these operations.—(*Oesterreichsche Zeitschrift für praktische Heilkunde*, 1859, 10–12.)

III.—*On the Treatment of Chorea by Cauterization.* By DR. HAMON.

Dr. Hamon has treated two cases of chorea with the potential cautery; in one of them the affection had assumed a very serious form, the convulsions involving nearly the whole body, and continuing unabated even during the night. The use of sulphur-baths, and the systematic administration of tartar-emetic, according to the method of Gillette and Bonfils, having produced no amelioration in the condition of the patient, Dr. Hamon applied amianth, soaked in concentrated nitric acid, to the side of the dorsal and lumbar parts of the spinal column, in such a manner that sixty small punctiform burns were produced at a distance of about one centimetre from each other, which healed in eight to twelve days without leaving any distinct cicatrix. On the evening of the same day the patient could already speak with greater ease, passed a comparatively quiet night, and the convulsions had lost much of their intensity. The operation was repeated twice, at intervals of several days, and effected a complete cure within three weeks, the disease having existed one month before the treatment was commenced.

In the second case, the disease presented a milder form, and affected only one side. After using sulphur-baths, without any benefit whatever, the patient, a girl sixteen years of age, was subjected to the treatment by cauterization; the cautery was applied only once, at eighty different points, and as in the former case, the symptoms were decidedly ameliorated the same evening.

The pain attending the operation is said to be very slight and transient; the œdema occurring in the commencement disappears very soon, and no suppuration takes place if the application is made but superficial.—(*L'Union Médic.*, No. xxx., 1859.)

IV.—*Professor Skoda's Formula in Scorbutus.*

Professor Skoda, of Vienna, employs the following preparation with advantage, associating it also with the other means generally had recourse to:—

Decoction of malt with fir sprouts, 275 parts; yeast, 25 parts; and syrup of orange-peel, 25 parts; a tablespoonful every two hours.—(*Bull. de Thérap.*, tome lvii., 1859.)

V.—*Saline Injections in Diphtheritis.* By M. ROCHE.

M. Roche states that he has been so successful in some cases in which he has tried the injection of a solution of chloride of sodium into the throat, that in his next case he is disposed to employ it as the sole means of treatment. He practices, in fact, a continuous, or almost continuous irrigation of the throat, by means of Eguisier's irrigator, provided with a canula having a very small jet. He believes that it is in such irrigations, whether employing salt, alum, or the chlorates, we should seek for curative agents.—(*Union Médicale*, No. 88.)

